# INFRASTRUCTURES OF CAREGIVING: RECONSTITUTING HOMELINESS AND FAMILY IN SINGAPORE

**DOI:** 10.1093/geroni/igad104.1216

**Published:** 2023-12-21

**Authors:** Arthur Chia, Rahul Malhotra

**Affiliations:** Duke-NUS Graduate Medical School, Singapore, Singapore; Duke-NUS Medical School, Singapore, Singapore

## Abstract

Caregiving comprises concrete everyday tasks that are built-on and revolve around the home, yet few studies have looked at caregiving as practice(s) that shape the home as a place of/for care. We examined the socio-material arrangements of people, activities, and things that enable caregiving at home, asking how caregiving is orchestrated, sustained, and delivered at home, and how the home is in turn re-constituted by caregiving. In-depth interviews were conducted with 40 family caregivers, aged 55-85 years, caring for their spouse and/or parent(s). Relations, activities, and things that enabled caregiving were analyzed through the theoretical lens of “care infrastructure”. Caregiving is enacted through a network of people and things – including family members, live-in hired helpers, medical and social workers; technologies like surveillance cameras and strategically placed mirrors at home to monitor care recipients; televisions turned-on 24/7; assistive devices such as wheelchairs and ramps, and placement of hospital beds at home, etc. – that change living arrangements and routines. Care tasks, responsibilities, and decisions are marked by negotiations, tensions, and compromises between people and with things. Caregivers appropriate, adapt, and improvise in situations of uncertainty, conflict, and impossible demand. We conclude that new socio-material arrangements of people, activities, and things that emerge because of caregiving change/disrupt the notion of home as a place of safety, familiarity, and comfort for caregivers. Understanding the emplacement/displacement and interconnection of things, activities, and people sheds light on how the home shapes the effects of caregiving and vice versa.

